# *Trypanosoma cruzi*-Derived Molecules Induce Anti-Tumour Protection by Favouring Both Innate and Adaptive Immune Responses

**DOI:** 10.3390/ijms232315032

**Published:** 2022-11-30

**Authors:** Teresa Freire, Mercedes Landeira, Cecilia Giacomini, María Florencia Festari, Álvaro Pittini, Viviana Cardozo, Alina Brosque, Leticia Monin, Valeria da Costa, Paula Faral-Tello, Carlos Robello, Eduardo Osinaga

**Affiliations:** 1 Laboratorio de Inmunomodulación y Vacunas, Departamento Inmunobiología, Facultad de Medicina, UdelaR, Gral Flores 2125, Montevideo 11800, Uruguay; 2 Laboratorio de Bioquímica, Departamento de Biociencias, Facultad de Química, UdelaR, Gral Flores 2124, Montevideo 11800, Uruguay; 3 Departamento de Inmunobiología, Facultad de Medicina, UdelaR, Gral Flores 2125, Montevideo 11800, Uruguay; 4 Laboratorio de Glicobiología e Inmunología Tumoral, Institut Pasteur de Montevideo, Montevideo 11400, Uruguay; 5 Laboratorio de Interacciones Hospedero-Patógeno, Institut Pasteur de Montevideo, Montevideo 11400, Uruguay; 6 Departamento de Bioquímica, Facultad de Medicina, UdelaR, Gral Flores 2125, Montevideo 11800, Uruguay

**Keywords:** lung cancer, glycosylation, anti-tumour immune response, innate immunity, *Trypanosoma cruzi*

## Abstract

Lung cancer remains the leading cause of cancer mortality worldwide. Thus, the development of strategies against this type of cancer is of high value. Parasite infections can correlate with lower cancer incidence in humans and their use as vaccines has been recently explored in preclinical models. In this study, we investigated whether immunisations with a *Trypanosoma cruzi* lysate from epimastigotes protect from lung tumour growth in mice. We also explore the role of parasite glycans in the induction of the protective immune response. A pre-clinical murine cancer model using the lung tumour cell line LL/2 was used to evaluate the anti-tumour potential, both in preventive and therapeutic settings, of a *T. cruzi* epimastigote-derived protein lysate. Immunisation with the parasite lysate prevents tumour growth and induces both humoral and cellular anti-tumour immune responses to LL-2 cancer cells. The induced immunity and tumour protection were associated with the activation of natural killer (NK) cells, the production of interferon-γ (IFN-γ) and tumour cell cytotoxicity. We also show that mannose residues in the *T. cruzi* lysate induce Toll-like receptor (TLR) signalling. The evaluated *T. cruzi* lysate possesses anti-tumour properties likely by activating innate and adaptive immunity in a process where carbohydrates seem to be essential.

## 1. Introduction

Non-small cell lung cancer (NSCLC) remains the leading cause of cancer mortality worldwide with a 5-year overall survival rate [1]. At present, the treatment of NSCLC mainly includes surgery, chemotherapy, radiotherapy, molecular targeted therapy and immunotherapy [2]. Immune checkpoint antibodies specific for CTLA-4 and PD-L1/PD-1 have shown remarkable clinical success in several cancers, including NSCLC patients [3]. However, only 20–25% of treated patients respond to checkpoint inhibition therapy [4]. In this scenario, the development of alternative immunological strategies is of high value. Cancer vaccines, including tumour antigen-associated vaccines, neoantigen-associated vaccines and cell vaccines, are designed to amplify tumour-specific immune cell responses via active immunisation [5,6]. Vaccination against lung cancer still remains a challenge, since it is non-immunogenic cancer and does not provoke profound immune responses [7] due to its ability to escape all the checkpoints and suppress immune responses by altering cell-mediated cytotoxicity [8,9].

Interestingly, parasitic infections can correlate with lower cancer incidence in humans. Indeed, a lower prevalence of cancer has been observed in patients with hydatid cyst disease [10] or chagasic megacolon [11,12]. In this line, our group has recently demonstrated that human cyst fluid can protect against lung tumours in a mouse preclinical experimental model [13]. Furthermore, infections by protozoan parasites such as *Trypanosoma cruzi* [14], *Toxoplasma gondii* [15,16,17] and *Plasmodium yoelii* [15,18] have conferred resistance to tumour growth in several animal models in a process that relies on the induction of anti-tumour immunity [16,17]. Indeed, it was found that malaria infection suppresses Lewis lung cancer growth via induction of innate and specific adaptive anti-tumour responses with the production of Th1-type cytokines [7]. Our group has previously shown that vaccination with a *T. cruzi* lysate from the epimastigote stage of this parasite induces anti-tumour immunity in two rat models that reproduce human carcinogenesis: colon cancer induced by 1,2-dimethylhydrazine and breast cancer induced by N-nitroso-N-methylurea [19]. Last, mice injected with splenocytes from animals immunised with *T. cruzi* lysate [20] showed a significant impairment of Ehrlich’s adenocarcinoma growth.

In the present study, in order to gain more insights into the anti-tumour immune response induced by *T. cruzi* antigens, we investigated whether immunisations with a *T. cruzi* epimastigote lysate protect from lung tumour growth in mice and explore the role of parasite glycans in the induced immunity. We demonstrate that immunisation with a *T. cruzi* epimastigote lysate induces strong immune responses against lung cancer in mice, both in preventive and therapeutic settings likely activating innate immunity in a process where carbohydrates seem to be essential.

## 2. Results

### 2.1. A Lysate from T. cruzi Epimastigotes Prevents Tumour Growth

**Figure 1:**
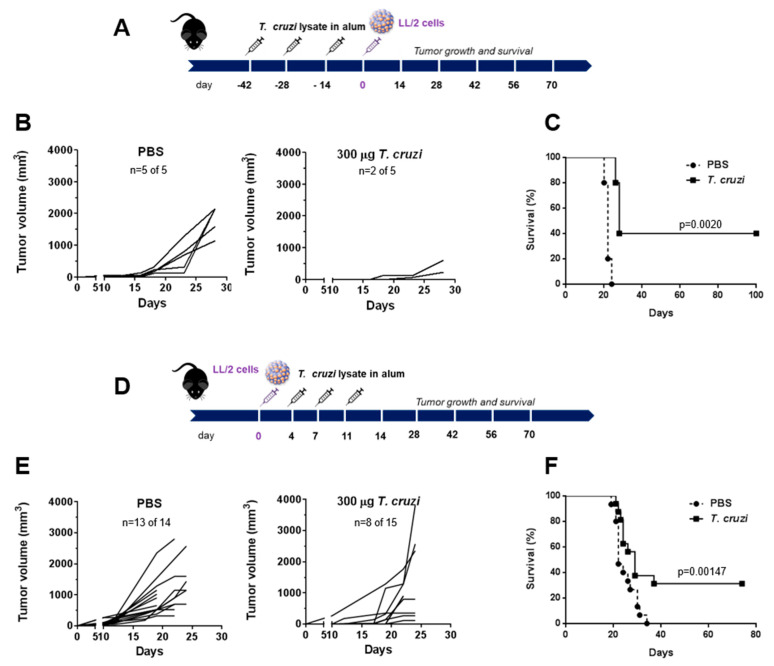
Immunisation with *T. cruzi* epimastigote lysate protects against LL/2 tumour growth in both prophylactic and therapeutic settings: (**A**) C57BL/6 mice were pre-treated three times in two-week intervals with *T. cruzi* (300 μg/mouse) or PBS in aluminium hydroxide (alum) before LL/2 cell s.c. challenge (100,000 cells/mouse). (**B**) Tumour growth was measured regularly using a calliper in mice that developed tumours; “n” represents the number of mice that developed tumours out of the total mouse number used in the experiment. (**C**) Survival of *T. cruzi*-pre-treated and control mice was followed for 100 days after tumour challenge. (**D**) After s.c. administration of LL/2 cells (day 0, 100,000 cells/mouse), C57BL/6 mice received *T. cruzi* (300 μg/mouse) or PBS in alum on days 4, 7, and 11. (**E**) Tumour growth was measured regularly using a calliper in treated and control mice. (**F**) Survival of *T. cruzi*-treated and control mice was followed for 80 days after tumour challenge. Representative results from three independent experiments are shown.

In order to analyse the ability of a lysate from *T. cruzi* epimastigotes to induce immune tumour protection, we carried out both prophylactic and therapeutic treatments in mice injected with the Lewis-type lung carcinoma cell line LL/2. In prophylactic settings, mice were pre-treated three times in two-week intervals with *T. cruzi* lysate or phosphate-buffered saline (PBS) in aluminium hydroxide (alum). Then, mice were challenged subcutaneously with LL/2 cells and tumour growth and mice survival were determined. Mice pre-treated with 300 µg of *T. cruzi* lysate did not show any tumours or presented small tumours (Figure 1A,B), which in turn, increased mice survival (Figure 1C). Furthermore, the protection (Figure 1D,E) and enhancement of mice survival (Figure 1F) inoculated with LL/2 cells were also demonstrated in a therapeutic setting, where mice were challenged with LL/2 followed by the treatment with the parasite lysate.

### 2.2. T. cruzi Epimastigote Lysate Administration Is Associated with Higher Activation of NK Cells in Tumour-Bearing Mice

**Figure 2:**
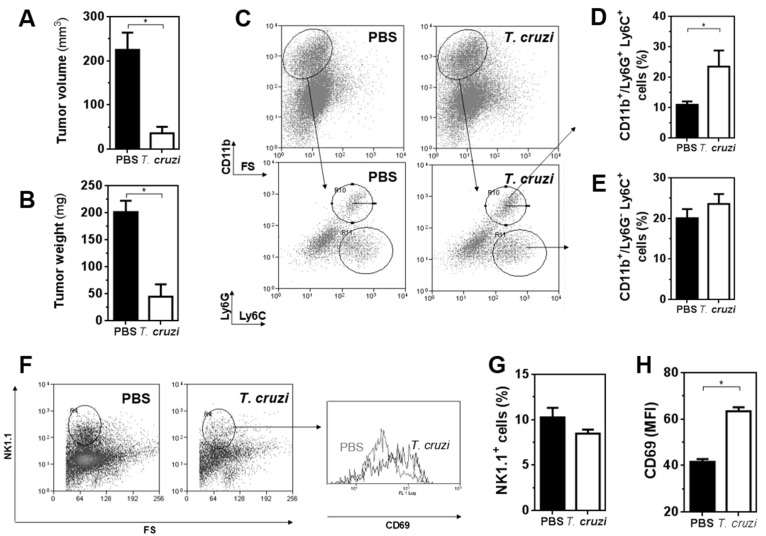
Analysis of the innate-associated cells in the tumour microenvironment of mice pre-treated with *T. cruzi* lysate and challenged with LL/2 tumour cells: (**A**) C57BL/6 mice were pre-treated three times in two-week intervals with *T. cruzi* or PBS in alum before LL/2 cells challenge (100,000 cells/mouse). On day 19, animals were sacrificed and tumours were removed. Tumours were either measured with a calliper (**A**) or weighed (**B**). Alternatively, tumours were stained either with anti-CD11b, Ly6G and Ly6C antibodies (**C**–**E**) or with anti-NK1.1 and -CD69 antibodies (**F**–**H**) and analysed by flow cytometry. (**C**) Gating strategy for CD11b^+^ cells. Frequencies of CD11b^+^/Ly6G^+^Ly6C^+^ (**D**) and CD11b^+^/Ly6G^−^Ly6C^+^ (**E**) cells. (**F**) Gating strategy for NK1.1^+^ cells. (**G**) Frequencies of NK1.1^+^ cells. (**H**) Expression of CD69 on NK1.1^+^ cells. Representative results from three independent experiments are shown (±SEM, indicated by error bars). Asterisks indicate statistically significant differences (* *p* < 0.05) Representative results from two independent experiments are shown.

To further study whether the administration of *T. cruzi* lysate in mice affected the innate immunity in the tumour microenvironment, we analysed different cell populations by flow cytometry from pre-treated and control mice that were sacrificed on day 19 after cell challenge (Figure 2). *T. cruzi*-treated mice presented significantly lower tumour volume (Figure 2A) and weight (Figure 2B) than control mice at the time of sacrifice. This was associated with an increase in the percentage of myeloid Ly6G^+^Ly6C^+^ cells, but not Ly6G^−^Ly6C^+^ in tumours from protected mice (Figure 2C–E). On the other hand, flow cytometry analyses showed no changes in the percentage of NK1.1^+^ cells in tumours of treated mice in relation to control mice (Figure 2F,G). Nevertheless, NK1.1^+^ cells from *T. cruzi*-pre-treated mice showed higher expression of CD69 than control mice (Figure 2F,H), indicating a higher level of activation.

### 2.3. T. cruzi-Treated Mice Present a Th1 Immune Response Characterised by Increased Cytotoxicity

**Figure 3:**
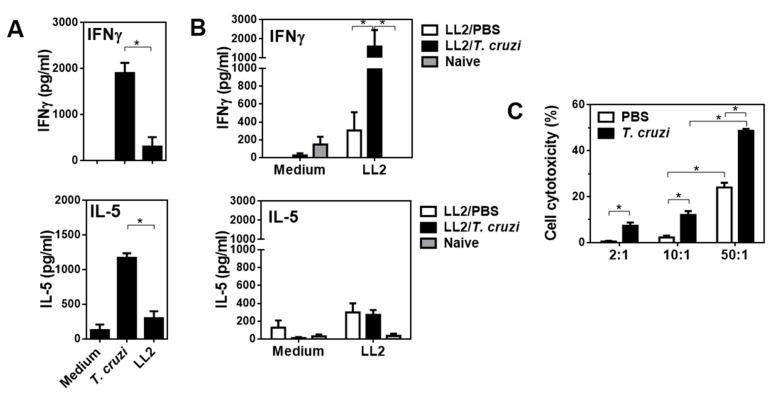
*T. cruzi*-treatment induces high production of IFN-γ by specific splenocytes with higher cytotoxic capacity: (**A**) Splenocytes (0.5–1 × 10^6^/well) from non-LL/2-tumour bearing mice immunised at days 4, 7 and 11 with *T. cruzi* and restimulated either with *T. cruzi* or LL/2 lysate (20 μg/mL). Spleens were collected on day 19 from sacrificed mice. (**B**) Alternatively, mice were challenged with LL/2 cells (100,000 cells/mouse) on day 0 and treated with *T. cruzi* lysate or PBS in alum on days 4, 7, and 11. Splenocytes from LL/2 tumour-bearing mice or naive mice were cultured in the presence or absence of an LL/2 lysate (20 μg/mL). Cultures were maintained at 37 °C for three days. Cytokine levels were measured in culture media by specific sandwich ELISA. (**C**) Splenocytes (effector cells, E) from tumour-bearing mice with or without *T. cruzi* treatment were cultured in presence of LL/2 cells (target cells, T). After overnight incubation at 37 °C, cells were stained with WST-8. Representative results from two independent experiments are shown (±SEM, indicated by error bars). Asterisks indicate statistically significant differences (* *p* < 0.05) by student *t*-test.

In order to further analyse the immune response induced by *T. cruzi* treatment, we evaluated the production of IFN-γ (as a readout of a Th1 response) and IL-5 (as Th2-like cytokine) by splenocytes derived from immunised mice after being restimulated with a lysate derived from LL/2 tumour cells. Splenocytes from *T. cruzi* immunised non-tumour-bearing mice produced high levels of both IFN-γ and IL-5 when stimulated with *T. cruzi*-derived molecules, although a very low non-significant production of these cytokines was obtained when restimulated with an LL/2 protein lysate (Figure 3A). In contrast, splenocytes from *T. cruzi*-treated and LL/2-inoculated mice induced higher levels of IFN-γ, but not of IL-5, than control mice injected with LL/2 cells (Figure 3B). The increased levels of IFN-γ of LL/2-restimulated splenocytes were associated with their increased in vitro cytotoxicity towards LL/2 tumour cells (Figure 3C), indicating that the cytotoxicity could be related to the presence of higher levels of IFN-γ and the presence of cytotoxic cells, like NK cells.

**Figure 4:**
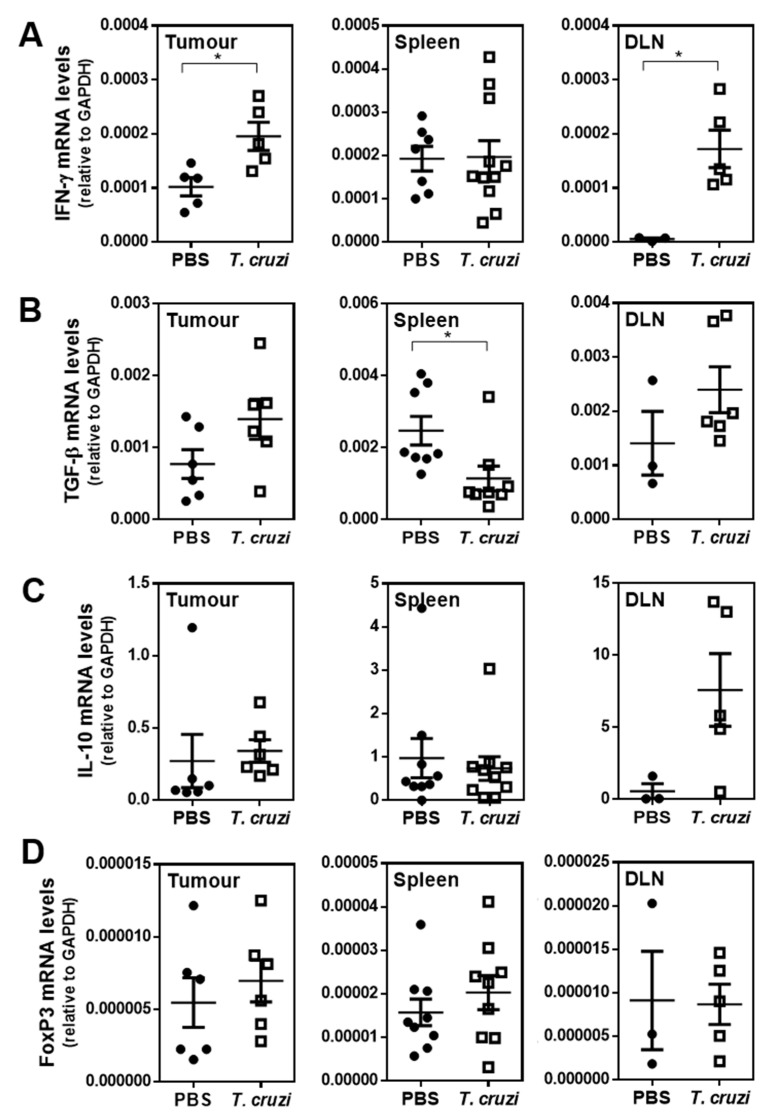
*T. cruzi*-treatment is associated with an increase in Th1-like cytokines and a decrease in Treg cytokines: IFN-γ (**A**), TGF-β (**B**), IL-10 (**C**) and FoxP3 (**D**) mRNA levels in tumours, spleens and DLN from *T. cruzi*-treated and control tumour-bearing mice were evaluated by quantitative RT-PCR. Results were expressed as the ratio between each evaluated cytokine and GAPDH expression. Representative results from two independent experiments are shown (±SEM, indicated by error bars). Asterisks represent statistically significant differences with * *p* < 0.05 by student *t*-test.

These results were supported by an increase in IFN-γ gene expression both in tumours and tumour-draining lymph nodes (DLN) from *T. cruzi*-treated mice (Figure 4A). On the contrary, a decrease in splenic TGF-β mRNA levels was associated with *T. cruzi* treatment (Figure 4B). Of note, no significant differences in the gene expression of IL-10 or FoxP3 were detected (Figure 4C,D).

### 2.4. T. cruzi-Treatment Induces IgG Antibodies That Recognise LL/2 Tumour Cells

**Figure 5:**
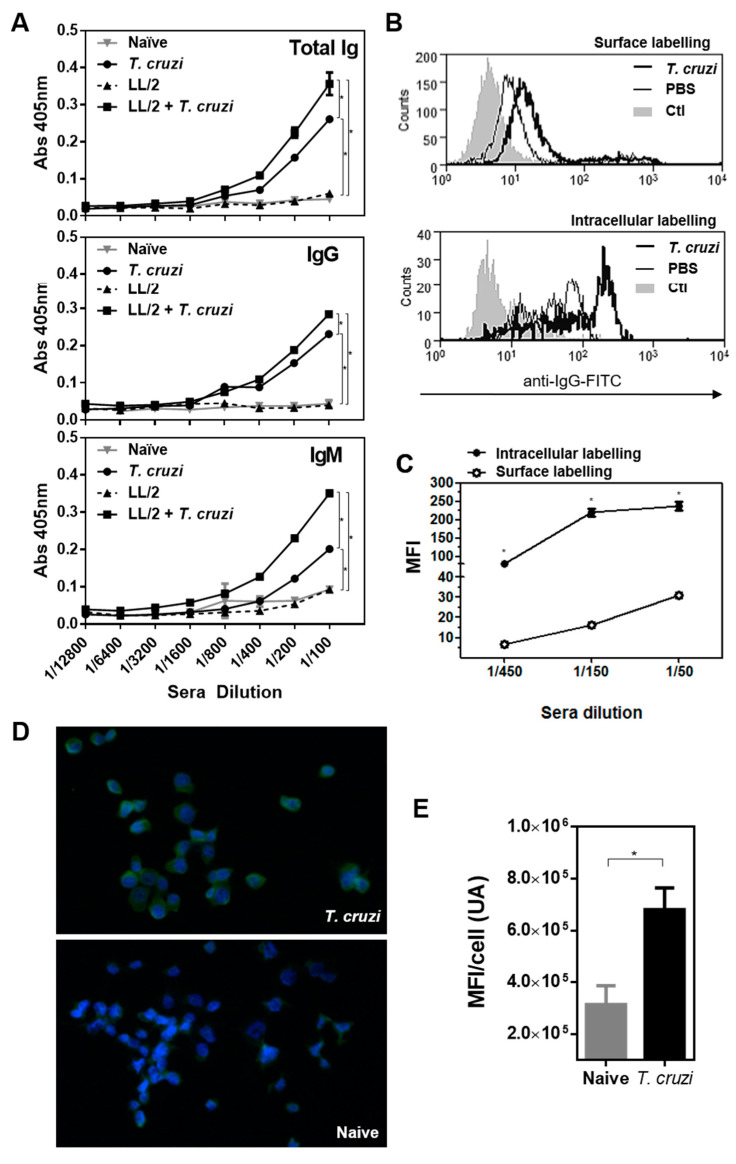
Antibodies induced after *T. cruzi* immunisation recognise tumour cells: (**A**) Antibody levels determined by ELISA in sera from *T. cruzi*-immunised and control mice injected or not with LL/2 tumour cells. (**B**) Histogram plots showing antibody recognition of membrane and cytosolic antigens on LL/2 cells. Flow cytometry analyses were carried out on permeabilised (intracellular staining) or non-permeabilised (surface staining) LL/2 tumour cells incubated with sera (diluted 1:100) collected from animals immunised with *T. cruzi* lysate. Controls consisted of sera from PBS-inoculated mice (PBS) or cells incubated with secondary antibody only (Ctl). Five thousand events were collected and gated on FSC vs. SSC dot plot. (**C**) Median fluorescence intensity (MFI) representing antibody recognition of surface and intracellular antigens on LL/2 cells, respectively. Different sera dilutions (1:50, 1:150, 1:450) were used, and MFI values were obtained after subtracting the corresponding pre-immune sera reactivity at the same dilution. (**D**) Antibody recognition of LL/2 cells by immunofluorescence (zoom 200×). (**E**) Antibody recognition of LL/2 cells by immunofluorescence was quantified with Image J software and represented as the mean fluorescence (MFI) per cell. Representative results from at least two independent experiments are shown (±SEM, indicated by error bars). Asterisks represent statistically significant differences with *p* < 0.05 by student *t*-test.

To analyse the induced anti-tumour humoral immune response, we evaluated whether antibodies induced by immunisation with *T. cruzi* lysate were capable of recognising tumour cells by flow cytometry. First, we analysed whether anti-*T. cruzi*-specific antibody titre changed in the presence of tumours. To this end, we evaluated *T. cruzi*-specific total, IgG and IgM antibodies in mice with or without tumours by ELISA. As shown in Figure 5, tumour-bearing mice immunised with *T. cruzi* produced higher levels of IgG and IgM-specific antibodies (Figure 5A). Then, we evaluated whether these antibodies were able to recognise LL/2 tumour cells, showing that. *T. cruzi-*induced IgG antibodies recognised both surface and intracellular molecules from tumour cells (Figure 5B). Furthermore, the recognition was antibody dose-dependent (Figure 5C). Finally, the capacity of *T. cruzi*-induced antibodies to recognise LL/2 tumour cells was confirmed by immunofluorescence (Figure 5D) and shown to be significantly higher than control antibodies (Figure 5E).

### 2.5. Glycans in T. cruzi Epimastigote Protein Lysate Mediate Tumour Protection

In order to establish whether glycan structures in the *T. cruzi* epimastigote lysate used in the study were involved in the immune response induced by the parasite lysate, we oxidised terminal carbohydrates with sodium periodate. This strategy is usually used to evaluate the functional roles of glycoconjugates [21].

**Figure 6:**
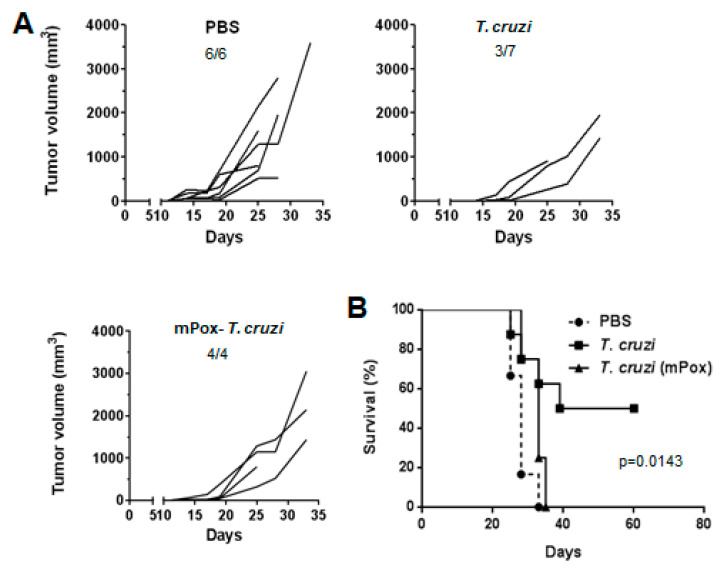
Glycoconjugates in *T. cruzi* lysate mediate tumour protection: (**A**) C57BL/6 mice (n = 4–7) were pre-treated three times in two-week intervals with *T. cruzi* (300 μg/mouse), m-periodate oxidised *T. cruzi* (mPox-*T. cruzi,* 300 μg/mouse) or PBS in alum before LL/2 cells challenge (100,000 cells/mouse). Tumour growth was measured regularly using a calliper. Numbers represent the number of mice that developed tumours out of the total mouse number used in the experiment. (**B**) Survival of treated and control mice was followed for 80 days after tumour challenge. Representative results from two independent experiments are shown.

During this process, the glycol groups in carbohydrates are oxidised to reactive aldehyde groups, which are in turn reduced with sodium borohydride [22]. Thus, the structure of carbohydrates is lost, as well as their possible biological activity. As depicted in Figure 6, mice pre-treated with oxidised *T. cruzi* lysate (mPox *T. cruzi*) developed a similar tumour size to those from the control group (PBS, Figure 6A). The role of glycoconjugates in anti-tumour protection was also confirmed when analysing mouse survival, since all tumour-bearing mice from the mPox-*T. cruzi* pre-treated group died before day 40, as control mice, while around 50% of mice injected with intact *T. cruzi* lysate presented prolonged survival (Figure 6B). Thus, carbohydrates from *T. cruzi* glycoconjugates seem to play a role in the induction of tumour protection.

### 2.6. The T. cruzi Epimastigote Lysate Contains Mannose-Rich Glycan Structures

**Figure 7:**
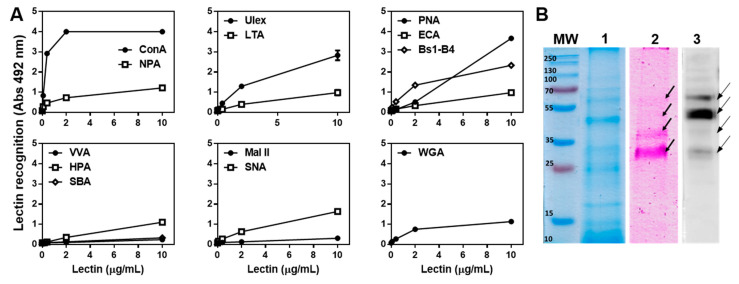
Glycoconjugates in *T. cruzi* lysate are recognised mainly by ConA: (**A**) Lectin recognition by Enzyme-linked Lectin assay. Reactivity by *Helix pomatia* lectin (HPA, specific for GalNAc), *Vicia villosa* lectin (VVL, recognising α/βGalNAc), *Sambucus Nigra* lectin (SNA, sialylated α2,6Gal), *Maackia amurensis* II lectin (MAL II, sialylated α2,3Gal),), *Dolichos biflorus* agglutinin (DBA, GalNAc), Soybean Agglutinin (SBA, α/βGalNAc), *Bandeiraea simplicifolia* lectin isolectin B4 (BS1-B4, αGalNAc), Peanut Agglutinin (PNA, with Galβ(1,3)GalNAc specificity), Wheat Germ Agglutinin (WGA, recognising GlcNAc in poly-*N*-acetyllactosamine repeats), Concavalin A (ConA, specific for Glc and Man in N-glycans), *Narcissus pseudonarcissus* agglutinin (NPA, αMan), *Ulex Europaeus I* (Ulex, α1,2-Fuc), *Lotus Tetragnolobus* agglutinin (LTA, αL-Fuc) was determined. (**B**) *T. cruzi* epimastigote lysate was run on 12% SDS polyacrylamide gels and stained either with Coomassie brilliant blue G-250 (lane 1) and with the GlycoPro glycoprotein staining kit (lane 2). Alternatively, proteins in gels were transferred to a nitrocellulose membrane and incubated with biotinylated ConA (0.4 μg/mL) (lane 3). Representative results from at least two independent experiments are shown. Arrows indicate several components visualised by the GlycoPro glycoprotein staining kit (lane 2) and by ConA recognition (lane 3).

Having established the relevance of parasite glycans in tumour protection, we characterised these glycan structures by using biotinylated plant lectins (Supplementary Table S1). A high reactivity with Concavalin A (ConA) and an intermediate reactivity with *Narcissus pseudonarcissus* agglutinin (NPA) revealed the presence of mannose-rich N-glycans. *Ulex europaeus I* (Ulex) also strongly reacted with the parasite lysate, which, together with *Lotus tetragnolobus* agglutinin (LTA) reactivity, indicated the presence of α-linked fucose residues (Figure 7A). Finally, the presence of Galactose-rich glycans was confirmed by reactivity detected with Peanut Agglutinin (PNA, with Galβ(1,3)GalNAc as its main ligand) and *Bandeiraea simplicifolia* lectin isolectin B4 (BS1-B4, specific for αGal) (Figure 7A). Interestingly, *Erythrina cristagalli* (ECA) recognition was rather low indicating that the Gal residues are not located in terminal β(1,4)lactosamine. Last, intermediate reactivity with *Sambucus nigra* lectin (SNA) demonstrated the presence of α-2,6-linked sialic acid residues. However, reactivity with GalNAc-specific lectins, such as *Vicia villosa* agglutinin (VVA), *Helix pomatia* agglutinin (HPA), Soybean Agglutinin (SBA), and Wheat Germ Agglutinin (WGA, recognising GlcNAc in poly-*N*-acetyllactosamine repeats) was significantly lower than the above-mentioned lectins (Figure 7A).

Considering that the ConA lectin highly reacted with glycans in the *T. cruzi* epimastigote lysate, we further characterised these glycoproteins. Figure 7B shows a protein profile of components in the parasite lysate stained with Coomassie blue (lane 1), with glycan-specific staining (lane 2) and with ConA (lectin blot, lane 3). ConA-reactive glycoproteins mainly comprised 4 components between 30 and 70 kDa.

### 2.7. Mannose-Rich Parasite Glycans Induce TLR-Signalling

**Figure 8:**
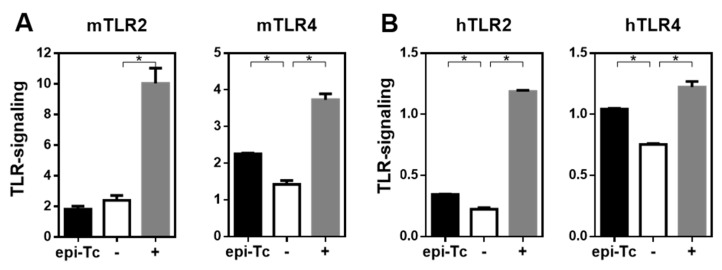
*T. cruzi* epimastigote lysate induces TLR-signalling: *T. cruzi* lysates were incubated with HEK-293 cells (50,000/well) expressing either murine TLR2 or TLR4 (**A**) and human TLR2 and TLR4 (**B**) overnight at 37 °C. Positive controls (+) consisted of LPS for TLR4 and in PAM3CSK for TLR2, while negative (−) control consisted of cultured cells in medium. Afterwards, the production of IL-8 or the quantification of phosphatase alkaline in cell culture was detected in the culture supernatant by specific sandwich ELISA or by direct absorbance measure with a spectrophotometer, respectively. Representative results from three independent experiments are shown (±SEM, indicated by error bars). Asterisks indicate statistically significant differences (* *p* < 0.05) by student *t*-test.

**Figure 9:**
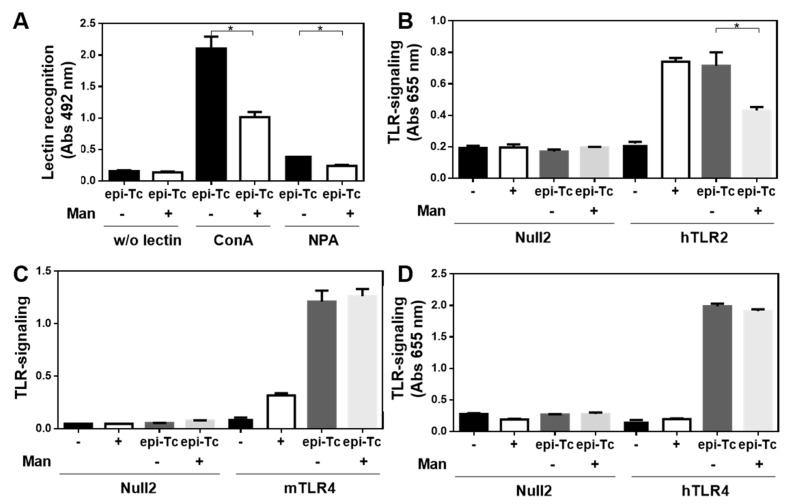
Mannose-rich glycoconjugates in *T. cruzi* lysate induce human TLR2 signalling: (**A**) ConA and NPA lectin recognition of *T. cruzi* lysates subjected to immobilised α-mannosidase. *T. cruzi* lysates subjected to immobilised α-mannosidase (Man) were incubated with HEK-blue-293 cells (50,000/well) expressing either human TLR2 (hTLR2, (**B**), mouse TLR4 (mTLR4, (**C**) and human TLR4 (hTLR4, **D**). Null2 consisted of control cells with basal endogenous TLR expression. Quantification of phosphatase alkaline in cell culture was detected in the culture supernatant by specific sandwich ELISA or by direct absorbance measure with a spectrophotometer. Positive controls (+) consisted of LPS for TLR4 and in PAM3CSK for TLR2, while the negative control (−) consisted of cells alone. Representative results from two independent experiments are shown (±SEM, indicated by error bars). Asterisks indicate statistically significant differences (* *p* < 0.05) by student *t*-test.

In order to analyse whether mannose-rich glycans present in the *T. cruzi* epimastigote lysate are capable of interacting with innate immune receptors, such as TLRs, we analysed their capacity to induce TLR-signalling in HEK-293 cells overexpressing either murine or human TLRs. As shown in Figure 8, the parasite components used in this study were capable of inducing both mouse and human TLR4 signalling, and human TLR2 signalling. To study whether mannose-rich glycans in the parasite lysate were involved in TLR induction, we specifically removed them by using immobilised α-mannosidase. As observed in Figure 9A, α-mannosidase successfully removed mannose residues, since a significant reduction in ConA reactivity was observed. Then, TLR signalling was studied both with the parasite lysate and with the lysate subjected to α-mannosidase. Interestingly, the removal of mannose residues from the *T. cruzi* epimastigote lysate significantly reduced human TLR2 signalling, although no changes were observed in both human and murine TLR4 signalling (Figure 9B,C).

## 3. Discussion

In this work, we demonstrate that the treatment with a *T. cruzi* lysate from the non-mammalian infective stage of the parasite, protects mice from tumour growth both in prophylaxis and therapy preclinical protocols. We characterised some aspects of innate and adaptive immunity that were associated with cancer protection. Animals injected with the parasite lysate presented a higher frequency of CD11b^+^/Ly6G^+^Ly6C^+^ cells in the tumours together with an increase in CD69 expression in NK cells, indicating a greater activation rate of these cells. The increase in granulocytes in tumours from *T. cruzi*-treated mice could account for neutrophils, which can have anti-tumour properties since they are capable of mediating tumour rejection [23] and preventing tumour growth [24] although their role in tumour immunity might be controversial [25]. On the other hand, NK cells associated with direct cytotoxicity can directly kill tumour cells, especially those that lack MHC class I molecules. Importantly, the density of NK cells in lung tumour tissue has been found to correlate positively with prognosis [26]. NK cell-associated direct cytotoxicity and cytokine production are crucial mechanisms for early innate host resistance against viruses, bacteria, or protozoa. In the same line, mice infected with *Plasmodium* stimulated activation of NK cells that killed lung cancer cells. In this context, the function of IFN-γ in inducing an increase in CD69 expression in NK cells was essential [27]. In addition, the infection with *N. caninum* [28], *Leishmania amazonensis* [29], and *T. gondii* [16] increased the levels of NK cells mainly through an IFN-γ-dependent pathway. Other works also describe the anti-tumour immunity induced by *Toxoplasma gondii* injection in melanoma preclinical models [16,17].

*T. cruzi* in vivo primed-splenocytes from tumour-bearing mice produced significantly higher amounts of IFN-γ when restimulated with the tumour LL/2 lysate, suggesting that the parasite lysate presents some common molecules with tumour cells. This Th1 cytokine was also increased in tumours and DLN from *T. cruzi*-treated mice. In addition to its role in activating NK cells, IFN-γ can induce tumour cell cycle arrest and establish tumour cell dormancy [30]. Considering the higher production of IFN-γ by tumour-bearing mice treated with the parasite lysate, the functional role of M1-like tumour-associated macrophages in the elicited immune response, such as tumour cell killing by reactive oxygen and nitrogen species, activating NK cells and promoting Th1 and cytotoxic immune responses [31], should be investigated. Further studies on the molecular and cellular immune response need to be carried out to deepen the understanding of the anti-tumour mechanisms induced by *T. cruzi* antigens. Other cell types, such as regulatory T or B cells and myeloid suppressor cells, should also be investigated. In this regard, the treatment with *T. cruzi* antigens was associated with a decrease in splenic TGF-β levels, suggesting an attenuation of a regulatory immune response. In fact, TGF-β favours tumourigenesis, metastasis, angiogenesis, autophagy and immune suppression and can suppress antigen-presenting function of macrophages and dendritic cells [32]. Last, it would be worth exploring whether the immune response induced by the parasite antigens eliminates tumours, by rechallenging protected mice with tumour cells. Indeed, in order to exploit the parasite antigens as therapeutic agents the analysis of the disease-free survival of treated mice together with a rechallenging with tumour cells would be essential.

Through flow cytometry, we observed that *T. cruzi*-induced specific antibodies recognised both membrane and intracellular molecules in lung cancer cells. In a previous work, we demonstrated that anti-*T. cruzi* antibodies were able to induce cancer cell death by antibody-dependent cellular cytotoxicity [19]. Alum, the primary adjuvant licensed for human use since 1920, promotes antibody and Th2 immune responses; likely by storing and allowing the slow release of antigens from the sites of immunisation [33]. Considering the role of this adjuvant in promoting inflammation and a potent antibody immune response, it would be worth studying whether the parasite lysate in the presence or absence of this adjuvant induces the production of IL-1β and IL-18. Furthermore, the immune response induced by the parasite lysate alone should eventually be evaluated.

Although the identification of the parasite molecules that induce the anti-tumour response remains to be elucidated, we can state that carbohydrates are essential for the induction of the immune response since the periodate-oxidation of carbohydrates present in the *T. cruzi* lysate abrogated its anti-tumour activity. Possible candidates include mucins (highly glycosylated cell surface proteins), highly relevant molecules in tumour biology and the generation of anti-cancer vaccines [34]. Others, such as TLR ligands, constitute attractive candidates to activate innate immunity. Furthermore, some protozoan parasites may help promote the maturation of dendritic cells since ligand molecules from parasites bind with Toll-like receptors (TLRs) to stimulate DC activation, such as glycosylphosphatidylinositol (GPI) from *Leishmania major* [35], *T. cruzi* [36], *P. falciparum* and *T. gondii* [37], and Profilin-like protein from *T. gondii* [38].

In this work, we demonstrate that some components from the *T. cruzi* lysate have anti-tumour properties and can trigger mouse and human TLR4 and human TLR2. We also show that ConA is one of the most reactive lectins with the used *T. cruzi* lysate, suggesting the presence, together with the reactivity with NPA, of highly mannosylated glycoconjugates. These glycans participated in hTLR2, but not TLR4, signalling, since their removal with α-mannosidase partially abrogated the *T. cruzi* lysate-induced hTLR2 signalling. Both TLR2 and TLR4 are expressed on the surface of innate myeloid cells, such as macrophages and dendritic cells, but also endothelial and epithelial cells, and are key initiators of the innate anti-tumour immune response [39]. In particular, TLR2 mainly interacts with lipoproteins. Interestingly, many of its ligands constitute mannosylated conjugates such as lipoarabinomannan, lipomannans and poshpatidylinisoitol dimannoside, among others [40]. It has been previously reported that TLR2 recognises glycosylphophatidylinositol from *T. cruzi* [36] and plays an important role in the immunity induced by *T. cruzi* infection in a process involving TNF and nitric oxide production by macrophages and inducing Th1-polarised immune responses [41,42], which could be in agreement with the induction of high levels of IFN-γ.

Altogether these results suggest that TLR2 ligands in the *T. cruzi* lysate could act as a natural adjuvant to induce anti-tumour responses. Indeed, TLR2 signalling plays an important role in the BCG-based treatment of bladder cancer [43] and in-transit melanoma [44]. Furthermore, TLR2 stimulation induced by a natural polysaccharide in a lung cancer model induces NK cell activation, proliferation, cancer cell-directed cytotoxicity, and the release of IL-2 and IFN-γ [45]. Moreover, TLR2 stimulation with the natural polysaccharide from medicinal mushroom *Trametes versicolor*, activates human NK cells, inducing IFN-γ secretion and the lysis of K562 leukaemia cells. In addition, this polysaccharide also enhances the efficacy of trastuzumab for the lysis of SKBR3 breast cancer cells favouring antibody-dependent NK cell cytotoxicity [46]. However, further experiments are necessary to confirm the role of glycans in the induction of tumour immunity by *T. cruzi* lysate through TLR signalling.

## 4. Materials and Methods

### 4.1. Cell Lines

Murine cancer cell line LL/2 (lung cancer, C57BL/6 background) was purchased at American Type Culture Collection (ATCC). Cells were cultured in RPMI, supplemented with 10% foetal bovine serum (FBS), 2 mM L-glutamine and 1 mM sodium pyruvate, in an incubator at 37 °C and 5% CO_2_. HEK293 cell lines stably transfected with mouse TLR2 and TLR4 derived from primary human embryonic kidney were purchased from Invivogen (San Diego, CA, USA) and cultured in high-glucose Dulbecco minimal essential medium supplemented with low-endotoxin 10% heat-inactivated FBS, GlutaMAX-I supplement, penicillin, streptomycin, and normocin at the recommended concentrations.

### 4.2. Mice

C57BL/6 female mice bred at the Biotechnology in Laboratory Animals Unit at the Institut Pasteur of Montevideo or URBE at Facultad de Medicina (UdelaR) were kept in an SPF environment and were used at 6–8 weeks of age. All the experimental protocols used in this work were approved by the Animal Experimentation Honorary Commission (CHEA) of the Universidad de la República (protocol number 070153-000809-18) and by the Ethics Commission of the Institut Pasteur of Montevideo (protocol number 009-12). Mouse handling, care, and experiments were carried out in compliance with institutional guidelines and regulations from the National Committee on Animal Research (Comisión Nacional de Experimentación Animal, CNEA, https://www.cnea.gub.uy/, accessed on 12 November 2021, National Law 18.611, Uruguay).

### 4.3. Preparation of T. cruzi Lysate

Epimastigotes of the Dm28 *T. cruzi* strain were grown at 28 °C in Liver Infusion Tryptose (LIT) medium, supplemented with 10% FBS. Parasites in the log phase were harvested and washed 3 times with PBS. They were then incubated in a lysis buffer containing 1% deoxycholic acid, 0.15 M glycine, 0.5 M NaCl, and pH 9.0 for 1 h at room temperature, 30 min at 37 °C and 30 min at 4 °C. The lysate was centrifuged at 20,000× *g* for 1 h at 4 °C to eliminate insoluble material and was stored at −80 °C. Endotoxin contamination of the *T. cruzi* lysate was evaluated using the Pyrochrome kit (Associates of Cape Cod Inc., Falmouth, MA, USA) according to the manufacturer’s instructions, obtaining between 0.01 and 0.4 UE/µg protein. which are in accordance with the amount of endotoxin allowed for inoculation in mice [47].

### 4.4. Oxidation of T. cruzi Lysate

The *T. cruzi* lysate was oxidised by incubation with 0.1 N HCl for 1 h at 80 °C, followed by neutralisation with NaOH. The resulting lysate was dialysed against 50 mM sodium acetate, pH 4.5 and was then incubated with 20 mM sodium m-periodate in acetate buffer for 1 h at room temperature, in the dark. The lysate was then dialysed against PBS. In order to control the integrity of lysate proteins, we performed Western Blot using a rabbit anti-cytosolic tryparedoxin peroxidase antibody. The band pattern obtained for the oxidised extract was compared to that of the original lysate as previously shown [13].

### 4.5. Evaluation of the Anti-Tumour Potential of the T. cruzi Lysate

C57BL/6 mice 6–8 weeks old were injected s.c. into the right flank with 1 × 10^5^ LL/2 cells diluted in PBS. In prophylactic experiments, mice were pre-treated three times (days 35, 21 and 7 before tumour cell challenge) with *T. cruzi* lysate (300 µg protein/mouse) in aluminium hydroxide (alum). In the therapeutic setting, mice were challenged on day 0 with 1 × 10^5^ LL/2 cells, and 4, 7 and 10 days later they were treated with *T. cruzi* lysate in alum. Control mice were treated with PBS in alum. The size of the tumour was calculated by the formula V (mm^3^) = (4/3) × pi × R_1_ × R_2_ × R_3_, where R_1_, R_2_, and R_3_ are the largest radio of the tumour in three dimensions. Mice were euthanised when the tumour diameter reached 20 mm or if they showed signs of distress. Survival of mice was followed for 90 days.

### 4.6. Evaluation of Cellular Immune Response

Proliferation assays were performed with cells from spleens from naive or tumour-bearing mice. Cells (0.5–1 × 10^6^ cells/mL) were cultured in a complete medium supplemented with 50 μM 2-mercaptoethanol (Sigma-Aldrich, St. Louis, MO, USA), in the presence or absence of an LL/2 lysate (20 μg/mL) or a *T. cruzi* lysate (20 μg/mL) at 37 °C and 5% CO_2_ for 3 days. IFN-γ and IL-5 levels were evaluated on culture supernatants by specific sandwich ELISA assays (BD Biosciences, Franklin Lakes, NJ, USA).

### 4.7. Evaluation of Humoral Immune Response

Mice or rabbits were immunised three times with *T. cruzi* epimastigote lysate (300 μg protein) in alum at two-week intervals. After the last immunisation, animals were bled and sera were evaluated by flow cytometry on the LL/2 cell line. Cells were first incubated for 15 min with sera (diluted 1:100) at 4 °C in PBS containing 2% foetal bovine serum and 0.1% sodium azide. Then, they were incubated for 15 min with an anti-mouse or ant-rabbit IgG goat antibody conjugated to FITC (Sigma, St. Louis, MO, USA). Alternatively, in order to evaluate sera recognition of intracellular antigens, cells were first permeabilised by incubating them in PBS containing 0.1% Triton-X100, 2% foetal bovine serum and 0.1% sodium azide. Paraformaldehyde-fixed cells were analysed on a CyAn ADP Analyser (Beckman Coulter, Brea, CA, USA), and analyses were performed with Summit V4.3 (Agilent, Santa Clara, CA, USA) to determine the mean fluorescence intensity (MFI). Alternatively, the recognition of LL/2 by anti-*T. cruzi*-antibodies was evaluated by indirect immunofluorescence microscopy. Slides were analysed in an Olympus IX81 epifluorescence microscope and analysed with the µManager 1.4.22 software. The quantification was performed with Image J 1.48v in 56 ± 16 cells/field.

### 4.8. Analyses of Immune Cells in Tumours by Flow Cytometry

Cells from tumours were washed twice with PBS containing 2% FBS and 0.1% sodium azide. Cells were then stained with anti-NK1.1 (PK136), -CD69 (H12F3), -CD8 (53-6.7), -CD11c (N418), -I-A/I-E (2G9), -CD11b (M1/70), -Ly6G (RB6-8C5), -Ly6C (HK1.4) and washed twice with PBS containing 2% FBS and 0.1% sodium azide and fixed with 0.1% formaldehyde. Cell populations were analysed using a CyAn ADP Analyser (Beckman Coulter). Antibodies were obtained from Affymetrix or Biolegend (San Diego, CA, USA).

### 4.9. Gene Expression by Quantitative RT-PCR

Spleen, DLN and tumour-derived cell suspensions were suspended in Tri-Reagent and RNA was purified. Then, the expression of different molecules was evaluated using an Eco real-time PCR System (Illumina, San Diego, CA, USA) using Fast SYBR^®^ Green Master Mix (Applied Biosystems, Waltham, MA, USA). Standard amplification conditions were 10 min at 95 °C for initial activation, followed by 40 thermal cycles of 15 s at 95 °C, 30 s at 60 °C and 30 s at 72 °C with a final extension of 10 min at 72 °C. Primers used were: *ifn-γ-*F: 5′-GGAGGAACTGGCAAAAGGATGGTG-3′; *ifn*-γ-R: 5′-GCGCTGGACCTGTGGGTTGT-3’; *tgf*-β-F: AACAATTCCTGGCGTTACCTT-3’; *tgf*-β-R: 5′-CTGCCGTACAACTCCAGTGA; *il-10*-F: 5′-TTCCCAGTCGGCCAGAGCCA-3′; *il-10*-R: 5’-GGGGAGAAATCGATGACAGCG-3′; *foxp3*-F: 5′-TCCAAGTCTCGTAAGGC-3′; *foxp3*-R: 5′-GCGAAAGTGGCAGAGAGGTA-3′; *gapdh*-F: 5′-ATGACATCAAGAAGGTGGTGAAG-3′; *gapdh*-R: 5′-TCCTTGGAGGCCAT GTAGG-3′. Results were expressed as the ratio between each gene under study and GAPDH expression. Expression was calculated using the 2−ΔΔCT method and normalised to GAPDH.

### 4.10. Lectin Recognition by Enzyme-Linked Lectin Assay (ELLA)

ELISA plates were coated with *T. cruzi* epimastigote lysate in 0.1 M carbonate buffer pH 9.6 overnight at 4 °C. Plates were then washed and blocked with 1% gelatine in PBS, followed by incubation with different concentrations of the following biotinylated lectins (Vector Labs, Newark, CA, USA, Supplementary Table S1): *Helix pomatia* lectin (HPA, specific for GalNAc), *Vicia villosa* lectin (VVL, recognising α/βGalNAc), *Sambucus nigra* lectin (SNA, sialylated α2,6Gal), *Maackia amurensis* II lectin (MAL II, sialylated α2,3Gal), *Dolichos biflorus* agglutinin (DBA, GalNAc), Soybean Agglutinin (SBA, α/βGalNAc), *Bandeiraea simplicifolia* lectin isolectin B4 (BS1-B4, αGalNAc), Peanut Agglutinin (PNA, with Galβ(1,3)GalNAc specificity), Wheat Germ Agglutinin (WGA, recognising GlcNAc in poly-*N*-acetyllactosamine repeats), Concavalin A (ConA, specific for Glc and Man in N-glycans), *Narcissus pseudonarcissus* agglutinin (NPA, αMan), *Ulex europaeus I* (Ulex, α1,2-Fuc), *Lotus tetragnolobus* agglutinin (LTA, αL-Fuc) in PBS containing 0.5% gelatine and 0.1% Tween-20. Lectin recognition was revealed by using horseradish streptavidin followed by o-Phenylendediamine dihydrochloride (OPD) incubation. Absorbance at 492 nm was determined with a plate spectrophotometer.

### 4.11. SDS-PAGE and Lectin Blot

Protein lysates were run on 12% SDS polyacrylamide gels in reducing conditions and stained either with Coomassie brilliant blue G-250 or with the GlycoPro glycoprotein staining kit (Sigma, St. Louis, MO, USA) according to the manufacturer’s instructions. Alternatively, proteins in gels were transferred to a nitrocellulose membrane (Amersham, France), blocked overnight with 3% BSA in PBS and incubated with biotinylated ConA (0.4 μg/mL) in PBS containing 1% BSA and 0.1% Tween-20. Afterwards, membranes were incubated with horseradish streptavidin and revealed with enhanced chemiluminescence (ECL) Western Blotting Detection Reagents (GE Healthcare, Milwaukee, WI, USA).

### 4.12. TLR-Signalling Assays

HEK293 cells (50,000/well) were incubated with *T. cruzi* lysate or TLR ligands as controls (LPS-EK, 50 ng/mL and Pam3CSK4, 20 ng/mL) overnight. Then, IL-8 was determined in culture supernatants by sandwich ELISA (BD Biosciences) as a readout of NF-kB activation. TLR2 and TLR4 signalling from humans were evaluated with HEK-Blue cells that contain an NF-κB-inducible alkaline phosphatase (AP) reporter gene system (Invivogen, CA, USA). Expression of secreted AP was detected using Quanti-Blue (Invivogen, CA, USA) and quantified by reading the absorbance at 620 to 655 nm by use of an ELISA plate reader.

### 4.13. Deglycosylation of T. cruzi Lysate with Immobilised α-Mannosidase

*T. cruzi* lysate demannosylation was performed using immobilised α-mannosidase from *Bacterioides thetaiotamicron* (Megazyme) prepared in our laboratory as previously described in Rodriguez et al. 2018 for the immobilisation of α-mannosidase from *Canavalia ensiformis* [48]. Briefly, 250 mg of agarose activated with cyanate esters group were incubated with 1 mL of α- mannosidase from *B. thetaiotamicron* (7.1 UE/mL; 2.7 mg/mL) in PBS for 4 h at room temperature under mild stirring. The supernatant was removed, and the immobilised enzyme was washed with MES buffer pH 6.5 containing 2.5 mM CaCl_2_ and stored at 4 °C.

500 µL of *T. cruzi* lysate (500 µg/mL) previously dialysed against PBS pH 6.5 were incubated with 250 mg of immobilised α-mannosidase (12 UE/g, 4.2 mg/g) under mild stirring at room temperature. Upon 24 h the immobilised α-mannosidase was removed from the reaction mixture by filtration, the lysate was then washed with MES buffer pH 6.5 containing CaCl_2_ 2.5 mM and stored at 4 °C until the next use. The supernatant was dialysed against PBS using membranes with a 3.5 kDa cut-off (Thermo Scientific, Waltham, MA, USA) in order to remove the released mannose. The corresponding controls were performed by incubating *T. cruzi* lysate with non-activated agarose under the same conditions. The demannosylated *T. cruzi* lysate was further used in lectin recognition and TLR signalling assays.

### 4.14. Statistical Analysis

The Student’s *t-*test was used to compare data from various experimental groups. A *p*-value < 0.05 was considered statistically significant. Mean and SEM are shown unless indicated otherwise. Survival was evaluated from the day of tumour injection until euthanasia, and the Kaplan–Meier test was used to compare mouse survival between the groups. Data were processed using GraphPad Prism 6.0 software.

## 5. Conclusions

In conclusion, we provide evidence of the anti-tumour effects of an epimastigote *T. cruzi* lysate in a lung tumour preclinical model in mice and describe the immune mechanisms that could participate in this anti-tumour protection. *T. cruzi* lysate induced both humoral (antibody) and cellular immunity, with the participation of innate immunity, especially NK cells.

## Data Availability

Not applicable.

## Supplementary Materials

The following supporting information can be downloaded at: https://www.mdpi.com/article/10.3390/ijms232315032/s1. References [49,50,51,52,53,54,55] are cited in the supplementary materials.
