# Supplementary material for: Trypanosoma cruzi-Derived Molecules Induce Anti-Tumour Protection by Favouring Both Innate and Adaptive Immune Responses
Source: Int J Mol Sci. 2022 Nov 30;23(23):15032. doi: 10.3390/ijms232315032 (PMC9739173; doi:10.3390/ijms232315032)
Supplement: Supplementary file 1 [file ijms-23-15032-s001.zip › ijms-1941548-supplementary.pdf]

International Journal of *Molecular Sciences*

MDPI

Article

Trypanosoma cruzi-Derived Molecules Induce Anti-Tumour Protection by Favouring Both Innate and Adaptive Immune Responses

Teresa Freire [1,*], Mercedes Landeira [1], Cecilia Giacomini [2], María Florencia Festari [1], Álvaro Pittini [3,4], Viviana Cardozo [4], Alina Brosque [4], Leticia Monin [4], Valeria da Costa [1], Paula Faral-Tello [5], Carlos Robello [5,6] and Eduardo Osinaga [3,4,*]

Supplementary Material

Table S1. Plant lectin primary and secondary specificity to different glycan structures.

Lectin	bbreviation	Primary specificity	Secondary specificity	Reference
Vicia villosa isolectine B4	**VVL**	Tn, α-GalNAc	β-GalNAc	[49]
Jacalin	**JAC**	Galβ(1-3)Gal-NAc, α-GalNAc	*Core* 3, sialyl-T	[50]
Helix pomatia agglutinin	**HPA**	α/ β-GalNAc		[51]
Glycine max agglutinin	**SBA**	α/β-GalNAc, Tn	Gal	[52]
Dolichos biflorus aggluti-nin	**DBA**	α-GalNAc, blood group A		[53]
Maackia amurensis II ag-glutinin	**MAL II**	NeuAcα(2-3)Gal		[54]
Sambucus Nigra agglutinin	**SNA**	NeuAcα(2-6)Gal	NeuAcα(2-6)GalNAc, Lac	[55]
